# Texas Beef

**Published:** 1875-08

**Authors:** 


TEXAS BEEF.—An English stock company was formed last winter in London for the establishment of a line of live stock steamers between that port and Galveston, Texas, for the shipment of beef cattle to British markets. The steamers, which have been fitted for the purpose of taking emigrants to Texas and cattle back to Great Britain, have commenced their trips. These are now loaded at Galveston with Texas cattle. This enterprise promises an extensive outlet for the immense herds of Texas cattle which have hitherto found markets only by long drives over the plains to our domestic markets, where they have been largely packed for foreign countries. An important influence on the cattle and provision markets may be anticipated, as well as upon the health of those who eat the meat. If, as we learn, the ships are well ventilated and the cattle properly cared for on the sea-voyage, the beef product will be far better than that got from badly-fed cattle driven by hurried marches, one or two thousand miles. This is the kind which the majority of beef eaters in New York have to depend upon, and it would be worth the while of some of our wise capitalists to consider whether they will not organize a line of vessels for conveying beef-cattle on the hoof from Texas to New York.

---

INFLAMMATION.—It is true that many wounds and bruises are relieved by exposing the injured part to the fumes of burning wool, or cloth made from wool, or pieces of hides, bones or hoofs, or leather, or other animal substance. The smoke of consuming animal substances has a remarkably antiseptic effect, and dangerous wounds from rusty nails have often been quickly relieved by it. Kindle a fire in an iron kettle and supply it with any old woolen rags, and then hold the sore place over the fire, as near as the heat will permit, for an hour or so. The heat will relieve the soreness, and so will the pungent smoke.

